# Insights Gained Into the Treatment of COVID19 by Pulmonary Surfactant and Its Components

**DOI:** 10.3389/fimmu.2022.842453

**Published:** 2022-05-03

**Authors:** Dan Li, Xianzheng Wang, Yingzhao Liao, Shouchuan Wang, Jinjun Shan, Jianjian Ji

**Affiliations:** ^1^ Jiangsu Key Laboratory of Pediatric Respiratory Disease, Institute of Pediatrics, Nanjing University of Chinese Medicine, Nanjing, China; ^2^ Department of Immunology, Nanjing University of Chinese Medicine, Nanjing, China; ^3^ Pediatrics of Traditional Chinese Medicine, Shenzhen Traditional Chinese Medicine Hospital, Shenzhen, China

**Keywords:** pulmonary surfactant, COVID-19, ARDS, therapeutic applications, respiratory viral infections

## Abstract

Pulmonary surfactant constitutes an important barrier that pathogens must cross to gain access to the rest of the organism *via* the respiratory surface. The presence of pulmonary surfactant prevents the dissemination of pathogens, modulates immune responses, and optimizes lung biophysical activity. Thus, the application of pulmonary surfactant for the treatment of respiratory diseases provides an effective strategy. Currently, several clinical trials are investigating the use of surfactant preparations to treat patients with coronavirus disease 2019 (COVID-19). Some factors have been considered in the application of pulmonary surfactant for the treatment COVID-19, such as mechanical ventilation strategy, timing of treatment, dose delivered, method of delivery, and preparation utilized. This review supplements this list with two additional factors: accurate measurement of surfactants in patients and proper selection of pulmonary surfactant components. This review provides a reference for ongoing exogenous surfactant trials involving patients with COVID-19 and provides insight for the development of surfactant preparations for the treatment of viral respiratory infections.

## Introduction

The ongoing coronavirus disease 2019 (COVID-19) pandemic, caused by severe acute respiratory syndrome coronavirus 2 (SARS-CoV-2), has affected over 200 million people worldwide (1). SARS-CoV-2 can induce lung injury that involves the airways, alveoli, and pulmonary vessels (2). Autopsies of patients with COVID-19 reveal patchy peripheral hemorrhage of the lung parenchyma, loss of alveolar elasticity (3), and fibrous cords with sticky secretion exuding from cut surfaces of the pulmonary alveoli, bronchi, and tracheae (3). Moreover, pathological examinations demonstrate diffuse alveolar damage, including inflammatory exudate, interstitial inflammation, and infiltrating monocytes, lymphocytes, and macrophages (4). Further, type II alveolar epithelial cell proliferation and focal desquamation of alveolar epithelial cells are observed (3). Severe COVID-19 is associated with multiple changes in immune profiles, affecting the ability of the host to mount a timely and effective immune response against SARS-CoV-2 (5). Eosinopenia and lymphopenia with a severe reduction in the frequency of CD4^+^ and CD8^+^ T cells, B cells, and natural killer (NK) cells are common features of patients with severe COVID-19 (5). Additionally, T cell lymphopenia driven by T cell sequestration in tissues or T cell apoptosis as a result of pro-inflammatory cytokines is common in patients with severe COVID-19 (6). Defects in type I IFN response are present in some patients with severe COVID-19 (7). Loss of function variants in loci that control toll-like receptor (TLR)3- and IRF7-dependent type I IFN immunity may lead to defects in type I IFN response in patients with severe COVID-19 (7). In addition, autoantibodies against IFN-α and IFN-ω are present in patients with COVID-19 (8), as well as substantial accumulation of activated immune cells, such as myeloid-derived suppressor cells (MDSCs) (5). Excess circulating immature monocytes, neutrophils, and myeloid progenitors—named emergency myelopoiesis—are almost pathognomonic features of severe disease (9). Circulating myeloid cells produce excessive amounts of inflammatory molecules, often causing a cytokine storm, which promotes multiple organ damage (9). In contrast, lung tissue–resident macrophages, such as alveolar macrophages, which are known to play an important role in tissue homeostasis and repair, are often depleted in patients with severe COVID-19 (10).

SARS-CoV-2 binds angiotensin-converting enzyme 2 (ACE2), which is expressed by pulmonary epithelial cells, causing acute interstitial pneumonia (11). Pulmonary epithelial cells can produce pulmonary surfactant, which contains a complex mixture of highly reactive compounds (12). Pulmonary surfactant covers the alveolar epithelium, facilitating breathing by reducing the surface tension of the air-water interface within alveoli, thereby preventing alveolar collapse and easing the mechanical work required to breathe (13). Emerging data indicate that pulmonary surfactant plays a pivotal role in the pulmonary host defense against respiratory viral infections, such as influenza and respiratory syncytial virus (RSV) infection (14). Moreover, pulmonary surfactant exerts anti-inflammatory and anti-viral effects against some respiratory viral infections (14–16). Recent studies show that SARS-CoV-2 infection may result in changes in pulmonary surfactant (14, 17). A study analyzing the lung transcriptome of patients with COVID-19 reported that the expression of surfactant proteins was downregulated during SARS-CoV-2 infection (18). Another study reported that surfactant protein production was deregulated in patients with COVID-19, resulting in increased expression of surfactant protein (SP)-A (19). A recent study indicated that levels of pulmonary surfactant lipids were markedly reduced in the bronchoalveolar lavage fluid of patients with COVID-19 compared to that in healthy controls (20). Moreover, SARS-CoV-2 infects alveolar type II cells (AT II cells) by binding to ACE2, thus impacting the production and turnover of pulmonary surfactant in AT II cells (14). Furthermore, SARS-CoV-2 infection may influence the recycling and catabolism of pulmonary surfactant in the alveoli by AT II cells and alveolar macrophages (14). These studies suggest that pulmonary surfactant is altered in patients with COVID-19, which not only influences surface tension-related properties but also impacts the host’s antiviral immunity following viral infection (13–15). Severe respiratory viral infection often causes a disorder of pulmonary surfactant in the lung, which increase the surface tension in the lung, and then induce alveolar collapse at end-expiration (14, 21). Supplemental pulmonary surfactant can reduce surface tension and prevent alveolar collapse, thereby preserving lung function for oxygenation (14). Therefore, pharmacological and therapeutic strategies aimed at readjusting pulmonary surfactant dysfunction during respiratory viral infection not only contribute to preserving lung function, but also inhibiting the pro-inflammatory response and limiting viral infection.

A previous study reported that intratracheal administration of surfactant resulted in improved lung compliance and less oxygen required to maintain acceptable oxygen saturation in RSV pneumonia (22). Moreover, administration of pulmonary surfactant has been used to effectively treat preterm infants with neonatal respiratory distress syndrome (NRDS), which is caused by pulmonary surfactant deficiency (23). Clinical data indicate that severe COVID-19 most commonly manifests as viral pneumonia-induced acute respiratory distress syndrome (ARDS), which is characterized by diffuse inflammatory damage that results in increased vascular permeability and reduced lung compliance (24). Interestingly, a recent study proposed that ARDS in COVID-19 resembled NRDS (17). Thus, some researchers have suggested that exogenous pulmonary surfactants may provide an effective treatment for COVID-19 (17, 25, 26). Accordingly, several studies have investigated the therapeutic value of administering exogenous pulmonary surfactants to patients with COVID-19 (17, 27, 28). Several clinical trials exploring surfactant preparations as a treatment for COVID-19 are ongoing using surfactants often used to treat NRDS (27, 28). To date, seven clinical trials have been conducted using exogenous surfactant preparations to treat patients with COVID-19 (27, 28) (**Table 1**). Although the outcomes of these trials have not yet been published, some initial results and case reports are available. One pilot study indicated that exogenous surfactant administration *via* bronchoscopy reduced the duration of mechanical ventilation and 28-day mortality rate of COVID-19, although the differences were not statistically significant (29). Further, a case report indicated that oxygenation was improved in a patient with COVID-19 after exogenous surfactant treatment (30). Despite these promising data, prior trials in which surfactant was administered to adults with ARDS were generally disappointing, with the majority showing no benefit (28). A meta-analysis of randomized controlled trials examining the effect of surfactant administration on adult patients with ARDS revealed no improvement in mortality or oxygenation (31). Therefore, the effectiveness of pulmonary surfactant for COVID-19 treatment remains unclear.

**Table 1:** Ongoing clinical trials of surfactant therapy for COVID-19.

NCT	Preparation	Conditions	Primary purpose	Intervention	Status
NCT04384731	CUROSURF^®^ (Poractant alfa)	COVID-19, ARDS	Design a new administration protocol for surfactant replacement therapy in adults, to be tested in COVID-19 adult ARDS patients.	Patient receiving surfactant (48 mg/kg) administered by bronchial fibroscopy. The total volume was divided in each of the five lobar bronchi.	Phase II
NCT04502433	CUROSURF^®^ (Poractant alfa)	COVID-19, ARDS	Evaluate the efficacy and safety of poractant alfa, administered by endotracheal instillation in adult hospitalized patients with SARS-COV-19 ARDS.	Three administrations with a 24 h dosing interval. Each endotracheal administration will consist of poractant alfa bolus: 30 mg/kg Lean Body Weight.	Phase II
NCT04375735	Bovine Lipid Extract Surfactant (BLES)	COVID-19, ARDS	Improve the mortality of mechanically ventilated COVID-19 patients. The primary goal is to first determine feasibility and safety.	BLES was administered in doses of 50 mg/kg ideal bodyweight, at a concentration of 27 mg/mL so a total volume of approximately 2 mL/kg was administered. The material was instilled *via* the suction catheter.	Phase II
NCT04389671	Lucinactant Sinapultide (KL4) Surfactant	COVID-19, Acute Lung Injury, ARDS	Evaluate whether Lucinactant can improve the acute lung injury and ARDS of COVID-19 patients.	Lucinactant administered as a liquid at a dose of 80 mg total phospholipids/kg Lean Body Weight.	Phase II
NCT04362059	COVSurf Drug	Respiratory Infections	Evaluate the delivery of surfactant in patient lungs using the COVSurf Drug Delivery System.	COVSurf Drug Delivery System.	Not Applicable
NCT04847375	Exogenous surfactant	COVID-19, ARDS	Evaluate effect of exogenous nebulized surfactant in the pre-intubation stages of the disease.	Nebulized surfactant was administered by face mask with a nebulizer.	Not Applicable
NCT04659122	AT-100 (rhSP-D)	COVID-19	Determine if an investigational drug, AT-100, is safe and tolerated by adults who have severe COVID-19.	Reconstituted AT-100 for intratracheal administration.	Phase I

The failure of surfactant preparations in treating adults with ARDS largely curtailed clinical interest in this approach over the last 15 years. However, the emergence of COVID-19-associated ARDS has generated renewed interest in this clinical approach. A recent review indicated that the success of surfactant therapy may be influenced by the dose administered, method of delivery, preparation utilized, mechanical ventilation strategy, and timing of surfactant treatment (28). Additional factors may need to be considered in the effective treatment of patients with COVID-19 using pulmonary surfactant. These factors are reviewed and discussed in this review.

## Accurate Measurement of Pulmonary Surfactant Is Necessary Before Administration

As previously mentioned, SARS-CoV-2 infection may induce changes in pulmonary surfactant. However, due to the complexity of the mixture, the extent of changes in each pulmonary surfactant component caused by SARS-CoV-2 infection remains unknown. Surfactant treatment in children with NRDS enabled infants to start producing endogenous surfactant, in part facilitated by re-utilization of surfactant constituents through recycling mechanisms (28, 32). Current evidence indicates that patients with COVID-19 have dysfunctional, rather than deficient pulmonary surfactant, and the dysfunction is not equivalent to a deficiency.

Pulmonary surfactant levels do not always decrease during viral respiratory infection. A study analyzing pulmonary tissue samples obtained from RSV-infected mice demonstrated changes in 86 surfactant lipids compared to control mice (Fold Change (FC) > 1.5 or FC < 0.67) (33). Among the altered lipids, some lipids displayed decreased abundance, such as diacylglycerols (DGs), triacylglycerols (TGs), and some palmitoylated phosphatidylglycerols (PGs), including PG 16:0_22:5 (FC=0.56) and PG 16:0_22:6 (FC=0.61). However, some lipids were more abundant, such as acylcarnitine (FC=3.77), phosphatidylinositol (PI) 18:0_18:2 (FC=2.53), lysophosphatidylinositol (lysoPI) 16:0 (FC=10.53), and some stearoylated PGs, including PG 18:2_20:4 (FC=10.84), PG 18:2_18:2 (FC=8.23), and PG 18:1_20:4 (FC=6.93) (33). During influenza virus infection, levels of phosphatidylcholine (PC), PG, and phosphatidylethanolamine (PE) in AT II cells were significantly lower in influenza-infected mice compared to those in control animals, while levels of phosphatidylserine (PS), PI, sphingomyelin, cholesterol, and DG were increased (34).

Moreover, levels of pulmonary surfactant proteins do not show a simple decreasing trend during viral respiratory infection. For example, one study reported that SP-A expression was significantly elevated whereas SP-B expression was unchanged in the lungs of patients with COVID-19 compared to those of control patients (19). The authors of this study suggested that the increased expression of SP-A, which was present in condensed masses inside the alveolar spaces, could invalidate the therapeutic efficacy of exogenous surfactant treatment (19).

Nevertheless, because it remains unclear whether pulmonary surfactant is deficient in patients with COVID-19, using pulmonary surfactant to treat COVID-19 may be unreasonable. Such attempts may put patients at risk, since the lungs of patients with severe COVID-19 are considerably damaged and highly susceptible to further injury (21). Additionally, the use of pulmonary surfactant to treat COVID-19 may further disturb the pulmonary microenvironment and aggravate lung burden. For example, due to pulmonary surfactant is associated with the sputum formation (35), administration of exogenous pulmonary surfactant may lead to the formation of sputum thrombi. In fact, previous trials with exogenous surfactant in patients with non-SARS-CoV-2-induced ARDS were unsuccessful (36), often because intervention took place when the lungs had already suffered irreparable damage (25). Thus, studies suggest that early use of exogenous surfactants is necessary for COVID-19 treatment (28).

Clarifying changes in pulmonary surfactant components during SARS-CoV-2 infection is needed before conducting related trials. Some researchers have suggested that pulmonary surfactants in patients should be assessed prior to initiating treatment (17). A point-of-care, rapid test that measures surfactant levels at birth has been developed for premature babies (37). This method may also be suitable for measuring surfactant levels in tracheal fluid obtained from patients with COVID-19. Other detection technologies, such as mass spectrometry, can also be used to measure surfactant components. In summary, accurate measurement and understanding of surfactant trends in COVID-19 may help determine the therapeutic application of pulmonary surfactants.

## Proper Selection of Pulmonary Surfactant Components

Pulmonary surfactant is a lipoprotein complex composed by weight of 80% phospholipids (PLs), 10% neutral lipids (mainly cholesterol), and 10% surfactant-associated proteins, named SP-A, SP-B, SP-C, and SP-D (14). The major PL components include PC (approximately 80%), PG (approximately 7–15%), and small quantities of PI, PE, and PS (approximately 5%) (14). The hydrophobic surfactant proteins SP-B and SP-C along with dipalmitoyl PC (DPPC) mainly confer surface tension–lowering properties to pulmonary surfactant (14). Meanwhile, the hydrophilic surfactant proteins SP-A and SP-D participate in pulmonary host defense and modify immune responses during respiratory viral infection (14). The host defensive functions of pulmonary surfactant components, including proteins and lipids, are summarized in **Table 2**.

**Table 2:** Roles of pulmonary surfactant components in viral infection.

Name	Function
SP-A	SP-A prevents influenza infection by occupying the HA binding site (38). SP-A limits RSV infection by binding the F and G protein (39). SP-A limits coronavirus infection by binding HCoV-229E virions (40). SP-A can neutralize SARS-CoV-2 through interaction with the S protein (41). SP-A mediates the phagocytosis of human papillomavirus 16 (HPV16) pseudovirions (42) and herpes simplex virus (HSV) in the host.
SP-D	SP-D can neutralize influenza virus through occupying the HA binding site (43). SP-D limits RSV infection by interacting with virus through attachment to the F and G proteins (44). SP-D limits coronavirus infection by binding HCoV-229E virions (40). SP-D limits SARS coronavirus by binding to the heavily glycosylated S protein (45). SP-D can neutralize SARS-CoV-2 through interaction with the S protein (41). rfhSP-D can compete with ACE-2 for binding to the S1 spike protein subunit of SARS-CoV-2 (16). SP-A can restrict HIV infection *via* binding to glycoprotein (gp)120 (46).
PC	DPPC can promote adenoviral entry into epithelial cells by binding the virus (47).
PS	PS can promote poxvirus infectivity (48), through apoptotic cell mimicry (49).
PG	1-Palmitoyl-2-oleoyl-sn-glycero-3-phosphatidylglycerol (POPG) can suppress RSV infection by binding to RSV with high affinity (50, 51). POPG can block influenza virus replication through inhibiting the attachment of influenza (52).
PI	PI can prevent RSV infection by preventing virus attachment to epithelial cells (53, 54). PI can reduce influenza propagation by binding to the virus with high affinity (54, 55).1-stearoyl-2-arachidonoyl-PI can defend against dengue virus infection (47).
PE	PE was required for the replication of a (+)RNA virus, such as tomato bushy stunt virus, hepatitis C virus, dengue virus, and West Nile virus (WNV) (56). RNA virus replication depends on PE enrichment at replication sites in subcellular membranes (57).
Cholesterol	Cholesterol promotes entry of many viruses into host cells (58), such as SARS-CoV (59), murine coronavirus (60), porcine deltacoronavirus (61), infectious bronchitis virus (62), Hepatitis C virus (63), Ebola virus (64), influenza (65), and so on.

SP-A and SP-D are known to protect against viral and other pathogenic infections by blocking the entry of numerous viruses, such as influenza, RSV, and human immunodeficiency virus (HIV), into host cells (16). SP-A and SP-D play roles in modulating coronavirus infection by binding to human coronavirus 229E (HCoV-229E) virions and preventing HCoV-229E from infecting host cells (40, 45). SP-A and SP-D can also bind to and neutralize SARS-CoV by interacting with the spike protein (45). Recombinant fragments of human SP-D (rfhSP-D) can compete with ACE-2 for binding to the S1 spike protein subunit of SARS-CoV-2, thereby reducing SARS-CoV-2 infection (66, 67). These results suggest that SP-A and SP-D may have therapeutic potential for the treatment of COVID-19.

Therapeutic pulmonary surfactants can be natural or synthetic (27). Natural pulmonary surfactants have been isolated from bovine, porcine, and human amniotic fluids (27). A previous study reported that natural (animal-derived) surfactants were more effective than synthetic surfactants (68, 69) because natural preparations contained all the surfactant phospholipids and hydrophobic proteins (SP-B and SP-C) needed to facilitate rapid formation of a functional surface film (28). However, the use of natural surfactants is accompanied by inherent risks, such as the transmission of infectious agents, immunogenicity, and impurities (70). Removal of highly immunogenic proteins such as SP-A and SP-D, terminal sterilization, and screening of animal sources have been used to minimize the potential risks (70). Interestingly, a previous study suggested that surfactant preparations containing SP-A and SP-D might have better efficiency (28). In contrast, completely synthetic surfactants possess a greater degree of chemical purity, thus avoiding some potential risks (70). Additionally, synthetic surfactants relieve the potential resource limitations of animal-derived surfactants, avoid religious factors, and have lower manufacturing costs (28). Theoretically, a synthetic surfactant could be formulated to contain SP-A and SP-D (28). Moreover, SP-B and SP-C are difficult to synthesize and synthetic surfactant preparations without these components display limited functionality (28). Studies suggest that synthesized forms and recombinant fragments of SP-A and SP-D may be feasible for therapeutic use (28, 67). Recombinant SP-D fragments have the advantage of smaller size, thus increasing the probability of reaching distal lung locations, and show higher resistance to proteases and collagenases than full-length SP-D (67). Therefore, synthesized forms and recombinant fragments of SP-A and SP-D may be considered in addition to natural pulmonary surfactants.

Pulmonary surfactant lipids also play a pivotal role in pulmonary host defense responses to respiratory viral infection (14). Recent studies reported that intranasal administration of some pulmonary surfactant lipids, such as PG and PI, prevented influenza and RSV infections (50, 51, 53, 54). PG and PI can markedly suppress RSV replication by binding to the virus with high affinity (50, 51, 53, 54). PG can block the replication of H1N1-PR8 and H3N2 influenza by binding to influenza virus with high affinity (52, 55). PI can prevent H1N1 spread from infected to non-infected cells in tissue culture by binding to H1N1 influenza with high affinity (52, 55). Further, plasmalogens can potentially be used as antiviral therapeutic and prophylactic agents against human cytomegalovirus (HCMV), influenza, WNV, and SARS-CoV-2 infections (71). Pulmonary surfactant lipids also exert anti-inflammatory effects against viral infection (72). For example, PC can inhibit multiple pro-inflammatory mediators to alleviate tissue damage (72), and DPPC inhibits LPS-induced pro-inflammatory cytokine secretion in airway epithelial cells and monocytes (72). Furthermore, PI and PG can inhibit pro-inflammatory cytokine responses in macrophages by blocking the TLR2 and TLR7 pathways. These studies suggest that pulmonary surfactant lipids may possess potential antiviral and anti-inflammatory properties against SARS-CoV-2 infection.

Not all pulmonary surfactant lipids protect against viral infection; some lipids facilitate viral infection (14). One study reported that PE was required for the replication of (+)RNA viruses, such as hepatitis C virus, dengue virus, and WNV (56). Further, the replication of some RNA viruses, such as tomato bushy stunt virus, depends on PE enrichment at replication sites in subcellular membranes (57). Cholesterol promotes the entry of several coronaviruses into host cells (58), such as SARS-CoV (59), murine coronavirus (60), porcine deltacoronavirus (61), and infectious bronchitis virus (62). Thus, cholesterol may contribute to coronavirus replication by acting as a key component in viral entry (73). Moreover, cholesterol may participate in the entry of other viruses into host cells. For example, Ebola virus glycoprotein interacts with cholesterol to enhance membrane fusion and cell entry (64), while hepatitis C virus replication depends on endosomal cholesterol homeostasis (63). Therefore, some cholesterol-lowering drugs, such as statins, can reduce viral infectivity (58). Statins may also serve as potential main protease inhibitors of SARS-CoV-2, thereby contributing to the control of viral infection (58). PS can promote poxvirus infectivity (48) through apoptotic cell mimicry (49). Some pulmonary surfactant preparations used for the clinical treatment of COVID-19 contain cholesterol, PE, or PS. Although there is no evidence that cholesterol, PE, or PS influence SARS-CoV-2 infection, this possibility should be considered before administering surfactants that contain these lipids.

The biological functions of some pulmonary surfactant components have been clarified. Therefore, components selected for surfactant preparation should refer to their biological functions. Additionally, further studies are warranted to explore the potential functions of other pulmonary surfactant components in order to guide appropriate selection for surfactant preparations.

## Conclusion

**Figure 1:**
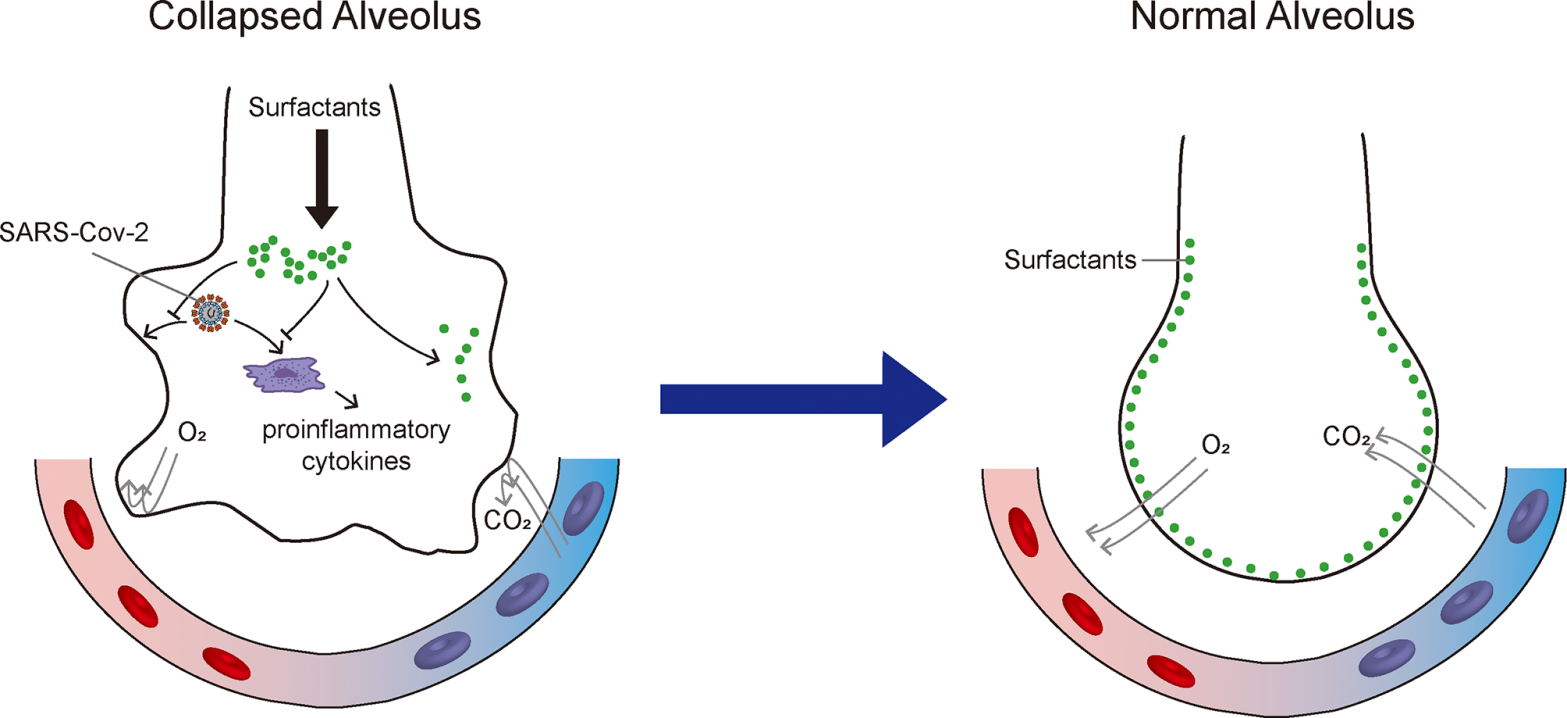
Therapeutic mechanisms of exogenous pulmonary surfactant for SARS-CoV-2 infection. Pulmonary surfactant can (1) mitigate acute respiratory distress syndrome by reducing alveolar surface tension and improving pulmonary mechanical properties; (2) suppress pro-inflammatory cytokine secretion; and (3) potentially inhibit SARS-CoV-2 replication and restrict SARS-CoV-2 infection.

The lung epithelium is constantly exposed to the environment and protected by pulmonary surfactant, which provides an important barrier against pathogen infection. Pulmonary surfactant prevents the dissemination of pathogens, modulates immune responses, and optimizes lung biophysical activity. Additionally, pulmonary surfactant may mitigate and reverse ARDS by reducing alveolar surface tension and improving pulmonary mechanical properties, while also exerting anti-inflammatory and antiviral effects (**Figure 1**). Thus, the application of pulmonary surfactant may provide an effective strategy for the treatment of respiratory diseases. This review highlights two new factors for consideration when selecting pulmonary surfactant therapy for COVID-19, namely accurate assessment of pulmonary surfactants in patients and appropriate selection of pulmonary surfactant components. This review provides a reference for ongoing trials investigating the use of exogenous surfactant in patients with COVID-19.

## Future Prospects

Pharmacological and therapeutic strategies to improve pulmonary surfactant dysfunction can prevent alveolar collapse at end-expiration, inhibit the pro-inflammatory response, and limit viral infection. Several clinical trials are currently exploring the use of surfactant preparations to treat COVID-19. In our opinion, accurate measurement of surfactants in patients and proper selection of pulmonary surfactant components should be considered prior to the clinical use of pulmonary surfactants. The rapid development of surfactant lipidomics has facilitated accurate measurement of pulmonary surfactants (33). Identifying pulmonary surfactant changes in patients with COVID-19 and modifying surfactant preparations accordingly can mitigate potential risks. Some components of pulmonary surfactant possess anti-inflammatory or antiviral properties and help prevent alveolar collapse, such as PG and SP-D (14, 16, 74). Several studies have reported that these components exert therapeutic effects against viral respiratory infection (14, 74). Clinical trial has been conducted to evaluate the safety and tolerated of AT-100 (rhSP-D) in patients with COVID-19 (**Table 1**). These studies suggest that a single lung surfactant component may effectively treat COVID-19. Moreover, using a single surfactant component may help avoid some potential risks. Taken together, this review provides important insight for the development of pulmonary surfactant preparations for the treatment of respiratory viral infections, including SARS-CoV-2.

## Data Availability Statement

The original contributions presented in the study are included in the article/supplementary material. Further inquiries can be directed to the corresponding authors.

## Author Contributions

JJ wrote the manuscript. XW and DL assisted with the manuscript preparation. SW, JS, and YL revised and polished the manuscript. All authors contributed to the article and approved the submitted version.

## Funding

This work was sponsored by the National Natural Science Foundation of China (82004204, 81774156), Qinglan Project of Jiangsu Province of China, Natural Science Foundation of Jiangsu Province of China (BK20210681), and Colleges and universities in Jiangsu Province Natural Science Research (21KJB310007).

## Conflict of Interest

The authors declare that the research was conducted in the absence of any commercial or financial relationships that could be construed as a potential conflict of interest.

## Publisher’s Note

All claims expressed in this article are solely those of the authors and do not necessarily represent those of their affiliated organizations, or those of the publisher, the editors and the reviewers. Any product that may be evaluated in this article, or claim that may be made by its manufacturer, is not guaranteed or endorsed by the publisher.

## Abbreviations

COVID-19, coronavirus disease 2019; SARS-CoV-2, severe acute respiratory syndrome coronavirus 2; ACE2, angiotensin converting enzyme 2; RSV, respiratory syncytial virus; AT II cells, alveolar type II cells; SP, surfactant protein; NRDS, neonatal respiratory distress syndrome; ARDS, acute respiratory distress syndrome; PI, phosphatidylinositol; PE, phosphatidylethanolamine; PG, phosphatidylglycerol; PC, phosphatidylcholine; PS, phosphatidylserine; PL, phospholipid; HIV, human immunodeficiency virus; HCoV-229E, human coronavirus 229E; rfh SP-D, Recombinant fragments of human SP-D; WNV, West Nile virus; HCMV, human cytomegalovirus; TLR, toll-like receptor; POPG, 1-Palmitoyl-2-oleoyl-sn-glycero-3-phosphatidylglycerol; DPPC, dipalmitoyl PC; lysoPI, lysophosphatidylinositol; DG, diacylglycerol; TG, triacylglycerol; MDSCs, myeloid-derived suppressor cells; NK, natural killer; TLR, toll-like receptor; FC, Fold Change; BLES, Bovine Lipid Extract Surfactant.
